# Social Environment and Hospitalisation after Release from Prison: A Prospective Cohort Study

**DOI:** 10.3390/ijerph14111406

**Published:** 2017-11-17

**Authors:** Alexander D. Love, Stuart A. Kinner, Jesse T. Young

**Affiliations:** 1 Melbourne School of Population and Global Health, The University of Melbourne, Parkville, Melbourne 3010, Australia; s.kinner@unimelb.edu.au (S.A.K.); jesse.young@unimelb.edu.au (J.T.Y.); 2 Centre for Adolescent Health, Murdoch Children’s Research Institute, Melbourne 3052, Australia; 3 Mater Research Institute-UQ, University of Queensland, Brisbane 4072, Australia; 4 Griffith Criminology Institute, Griffith University, Brisbane 4222, Australia; 5 School of Public Health and Preventive Medicine, Monash University, Melbourne 3800, Australia; 6 Centre for Health Services Research, School of Population and Global Health, The University of Western Australia, Perth 6009, Australia; 7 National Drug Research Institute, Curtin University, Perth 6008, Australia

**Keywords:** hospitalization, health equity, health priorities, health services, environmental health, population health

## Abstract

This study examined the association between remoteness and area disadvantage, and the rate of subsequent hospitalisation, in a cohort of adults released from prisons in Queensland. A baseline survey of 1267 adult prisoners within 6 weeks of expected release was prospectively linked with hospital, mortality and reincarceration records. Postcodes were used to assign remoteness and area disadvantage categories. Multivariate Andersen–Gill regression models were fitted to test for associations between remoteness, area disadvantage and hospitalisation after release from prison. Over a total of 3090.9 person-years of follow-up, the highest crude incidence rates were observed in areas characterised by remoteness and area disadvantage (crude incidence rate (IR) = 649; 95%CI: 526–791), followed by remoteness only (IR = 420; 95%CI: 349–501), severe area disadvantage only (IR = 403; 95%CI: 351–461), and neither of these factors (IR = 361; 95%CI: 336–388). Unadjusted analyses indicated that remoteness (hazard ratio (HR) = 1.32; 95%CI: 1.04–1.69; *p* = 0.024) was associated with increased risk of hospitalisation; however, this attenuated to the null after adjustment for covariate factors. The incidence of hospitalisation for those who live in remote or socio-economically disadvantaged areas is increased compared to their counterparts in more urban and less socio-economically disadvantaged areas. Experiencing both these factors together may compound the hospitalisation in the community.

## 1. Introduction

In most countries, including Australia, growth in the prison population is outstripping population growth [1,2]. In Australia, people in prison have relatively good access to healthcare, but many face an interruption in care after release from custody. Individuals must then quickly adapt to managing their own healthcare needs and navigating multiple, poorly coordinated healthcare systems. People released from prison are often profoundly disadvantaged; typically, they are less wealthy, have poorer educational attainment, are more likely to be homeless [3] and are less likely to be employed than the general population. In the context of escalating health risk behaviours and often inadequate transitional healthcare, many individuals experience deterioration in health after release from prison; and any health gains made in prison are rapidly lost [4,5,6]. Accordingly, compared to the broader Australian population, people released from prison are at increased risk of mental illness, substance use disorder, infectious disease, injury and chronic disease [7,8,9]. In many cases, these conditions co-occur and interact in a synergistic fashion [10]. The number of deaths soon after release from prison is markedly higher than the number in prison, providing a stark illustration of the rapid escalation in health risk after release from custody [11]. Many of these deaths are from preventable causes.

People released from prison are also at increased risk of hospitalisation, an objective measure of poor health outcomes [7,12]. Hospitalisations are generally more expensive than preventative health measures and other health services such as general practitioner (GP) contact, and often hospitalisations are ambulatory-care sensitive and thus preventable [12]. Individuals with pre-existing mental health conditions appear particularly vulnerable to experiencing poor health outcomes after release from custody [13]. Prior research in Australia has found that the majority of hospitalisations in the year after release from prison occur to treat mental and behavioural disorders or injuries [7].

There is a growing body of research that has examined the relationship between the environment and human health in Australia, the majority of which focuses on interactions between the physical environment and health (for example, the impact of air pollution on cardiovascular disease [14]; the impact of urban sprawl on obesity [15]; and the impact of perceived neighbourhood ‘greenness’ on mental health [16]. In Australia, remoteness and area disadvantage may be two important social–environmental determinants of health which play a role in determining health outcomes for people recently released from prison.

Remoteness is defined by where a person is situated relative to population centres and essential services, and certain health outcomes may result from this situation. For example, urban density may predict exposure to some health risks such as air pollution, a factor with known health consequences [14]. Conversely, remote areas may be associated with some health risks, for example decreased access to health services, particularly specialist services [17]. This is particularly relevant for Indigenous Australians, a disproportionate number of whom live outside metropolitan areas [18]. 

Area disadvantage refers to the extent to which people in a specific area are unable to participate in economic, political and social aspects of life, and is largely influenced by poverty, deprivation and social exclusion [19]. Although not necessarily an indicator of an individual’s personal circumstances, area disadvantage describes some aspects of what a person is exposed to from living in a particular area. For example, an increased number of overcrowded dwellings in an area may predict increased crime rates, which may in turn influence health outcomes for everyone living in that area. Over-crowdedness is related to low individual socio-economic status (SES); however, living in an area characterised by a high rate of overcrowded dwellings may have consequences for people living in that area above and beyond their individual SES [20]. People released from prison often live in more disadvantaged areas and experience disproportionately high rates of housing instability. Therefore, people released from prison may be particularly vulnerable to any effect area disadvantage may have on health outcomes. Further, negative stigma associated with incarceration may transfer to areas with a high density of ex-prisoners, which then in turn may negatively impact individuals’ economic and social opportunities, as well as health outcomes [21]. 

Indigenous Australians, an already disadvantaged group in terms of health outcomes [18,22,23,24,25], are over-represented in prison by an age-adjusted factor of 13 compared to their non-Indigenous counterparts [26]. Indigenous Australians experience unique health inequities, social determinants and barriers to health service access [27,28]. Previous research suggests that Indigenous people released from prison are a particularly vulnerable group and may experience worse health outcomes compared to non-Indigenous people [25,27,29,30,31]. Indigenous Australians experience higher rates of mental illness, which have been associated with social disadvantage and with living outside major metropolitan areas [32].

Studies of the relationship between remoteness and health have produced mixed findings. In some studies, remoteness predicts poorer health outcomes (for example, see [14,17]), whilst other findings indicate that prevalence of mental illness in Australia does not differ significantly between major cities and more remote areas [33,34,35]. This discrepancy is likely due in part to varying definitions of remoteness between countries and between studies. Previous findings suggest that both remoteness and area disadvantage negatively impact on health outcomes, and people released from prison are more likely to reside in disadvantaged and/or remote areas [36]. As such, returning to these social environments may compound the poor health outcomes experienced by people who transition from prison to these environments. However, to our knowledge, no studies have examined the relationship between remoteness and health outcomes for people released from prison. 

Similarly, the effect of area disadvantage on health outcomes after release from prison is currently unknown. Previous research in Australia has observed a positive association between area disadvantage and both smoking [37] and psychological distress [38]. Although the relationship between area disadvantage and health outcomes has yet to be examined in people released from prison, area disadvantage has been shown to predict some poorer health outcomes amongst Indigenous Australians, a disadvantaged population in Australia who are markedly over-represented among those who experience incarceration [26,39]. 

The aim of this study was to examine the association between remoteness and area disadvantage, and the rate of subsequent hospitalisation, in a cohort of adults released from prisons in Queensland, Australia. We hypothesised that remoteness and area disadvantage would be associated with an increased incidence and increased risk of hospitalisation, after adjusting for confounding. A secondary aim was to consider whether the association between these environmental factors and hospitalisation differed for Indigenous and non-Indigenous people. 

## 2. Materials and Methods

### 2.1. Study Population

We used cohort data from the Passports study, a randomised controlled trial of a case management intervention for adults (≥18 years) transitioning from prison to the community, described in detail elsewhere [4,40,41]. Briefly, participants were sentenced adult prisoners within six weeks of expected release from seven prisons in Queensland, Australia, interviewed between 1 August 2008 and 31 July 2010. The seven prisons from which participants were recruited included four in the densely populated south-east region of Queensland and three in the less densely populated far north of Queensland. These prisons were identified by Queensland Corrective Services as those from which the majority of sentenced prisoners were released in the State. The study cohort included people released from prison from every security classification: maximum, high and low. Queensland is the second largest state in Australia by area (covering 1.85 million km^2^) and the third largest by population (approximately 4.9 million people). This very large state is characterised by several large population centres in the south-eastern corner, and large rural and remote areas in the northern and western parts of the state. Queensland’s state capital, Brisbane, is the third largest metropolitan area in the country and accounts for almost half of the State’s population. Some communities in Queensland are located hundreds of kilometers away from major population centres, where most health services are located.

With the exception of intentionally over-sampling women, the Passports study was found to be broadly representative of adults being released from Queensland during the recruitment period [40]. Participation in this study was voluntary. All individuals were screened by trained researchers for the capacity to provide informed consent prior to being invited to participate. Individuals who did not demonstrate this capacity were not invited to participate. All individuals who elected to participate provided written, informed consent for participation.

### 2.2. Baseline Measures

A structured interview was administered by a trained researcher and assessed a range of socio-demographic and health measures, including current health status based on self-reported information and results of validated screening tools, and expected future living circumstances. Baseline interviews took place prior to randomisation. 

Socio-demographic measures included sex, age, Indigenous status (Aboriginal and/or Torres Strait Islander vs. neither), employment (employed vs. not employed prior to incarceration), accommodation (stable vs. unstable prior to incarceration), and education (≥10 years vs. <10 years of schooling). Self-reported juvenile detention (had experienced juvenile detention vs. had not experienced juvenile detention) was included as a justice-related characteristic. Participants also identified the postcode they expected to reside in after release from prison, which was used to define area disadvantage and remoteness (detailed below). 

Baseline health measures were assessed using validated screening tools including the Short Form 36 Health Survey (SF36 version 2) [42], a measure of health-related functioning that produces a Physical Health Component Summary Score and a Mental Health Component Summary Score; the Fagerström test for nicotine dependence [43], which measures the extent to which a person is nicotine dependent; the Patient Activation Measure (PAM) [44], a valid measure of the ability to self-manage one’s health; and the ENRICHD Social Support Inventory [45], which measures one’s level of perceived social support. In addition, we included self-reported lifetime history of injecting drug use (IDU) and study group for the original randomised control trial, as covariates.

### 2.3. Ascertainment of Social–Environmental Factors

Remoteness data were obtained from Accessibility/Remoteness Indexes of Australia (ARIA), a group of indices that rank and categorise areas according to road distances from services in Australia [46]. The use of ARIA data to measure remoteness is consistent with previous health research in Australia [3,47]. Remoteness categories include major cities, inner regional areas, outer regional areas, remote areas, and very remote areas [48]. ARIA categories were collapsed to a dichotomous variable: major cities and inner-regional areas, versus outer-regional, remote and very remote areas.

Area disadvantage data were obtained from the Australian Bureau of Statistics’ Socio-Economic Indexes For Areas (SEIFA) [49], a group of indices that rank areas according to various socio-economic indicators [19,49]. This research utilised the Index of Relative Socioeconomic Disadvantage (IRSD), which ranks all areas within Australia according to relative socio-economic disadvantage. The use of the IRSD is consistent with prior research that has looked at area disadvantage and health in an Australian context (for example, see [50]). We categorised postcodes within the most disadvantaged quintile as ‘severe area disadvantage’ while postcodes within the four less disadvantaged quintiles were categorised as ‘not severe area disadvantage’.

### 2.4. Data Linkage

We used probabilistic linkage to match baseline data with state-wide hospital records [51] and mortality records from the National Death Index (NDI) [52]. To ensure all state-wide health records were collected, probabilistic linkage was conducted using all known aliases for participants, which has been shown to increase the sensitivity of linkage without adversely affecting specificity [53]. We used deterministic linkage to link baseline data with reincarceration records from Queensland Corrective Services (QCS) [54]. Linked hospital data were obtained for the period 1 July 1999 to 31 July 2012, which is the end of the study period. 

Pre-incarceration hospitalisation that occurred for any reason between 1 July 1999 and date of incarceration (hospitalised vs. not hospitalised) was ascertained from the linked hospital data.

### 2.5. Ascertainment of Outcome

The outcome of interest was hospitalisation in Queensland after release from prison. In instances where participants were reincarcerated, hospitalisations that occurred during incarceration were excluded, as prisons are controlled environments which are not subject to the environmental exposures that are the focus of the current study. In order to examine potential differences in the causes of hospitalisation and related expenditure by remoteness and area disadvantage, we calculated the five most common causes of hospitalisation, and associated hospital bed days, according to remoteness and area disadvantage.

### 2.6. Statistical Analysis

Descriptive statistics were calculated for all variables. Chi-square and independent samples *t*-tests were used to test differences according to remoteness and area disadvantage for categorical and continuous covariates, respectively. 

The crude incidence rate of hospitalisation per 1000 person-years after release from prison was determined for the total cohort and according to remoteness and area disadvantage. Time at-risk was defined as the time between date of initial release from prison and date of death or the end of the study observation period (31 July 2012), whichever came first. Time at-risk was censored at date of reincarceration in instances where participants were not re-released prior to the end of the study period. If participants were reincarcerated and subsequently released prior to the end of the study observation period, time at-risk recommenced. Only hospitalisations that occurred during time spent in the community were included in the analysis.

To estimate the association between remoteness, area disadvantage, covariate factors and hospitalisation after release from prison, we fit univariate and multivariate Anderson-Gill regression models, a modified Cox proportional hazards model that accounts for recurrent events [55]. Covariates were included if they were found to be potentially associated with either remoteness or area disadvantage (*p* < 0.1) from the Chi-square analyses and *t*-tests, or in instances where a confounding relationship was theoretically plausible. Because all participants were part of a randomised controlled trial (RCT) where they were randomised to receive either personalised case management or usual care [40], we tested the association between RCT randomisation group and remoteness, area disadvantage and hospitalisation, all of which were non-significant (all *p* > 0.05). Therefore, the RCT randomisation group was not included in the final model in the interest of model parsimony. The multivariate model was adjusted for living in rural or remote areas, severe area disadvantage, being female, age, being Indigenous, being employed prior to incarceration, having stable accommodation prior to incarceration, having completed at least 10 years of school at baseline, injecting drug use, hospitalisation prior to incarceration, high smoking dependence, scoring above cohort median on Patient Activation Measure, level of social support, physical health score and mental health score. To examine the potential modifying effect of Indigenous status, remoteness and area disadvantage, we fit an additional multivariate model including two-way interaction terms between Indigenous status and remoteness, Indigenous status and area disadvantage, and remoteness and area disadvantage. All tests were two-tailed with significance set at *p* < 0.05.

To determine the most common reasons for hospitalisation, we categorised the number of hospitalisations and hospital bed days according to ICD-10 primary chapters, according to the principal diagnosis for each hospitalisation [56]. We report the five most common causes of hospitalisation stratified by remoteness and area disadvantage.

## 3. Results

Of 1325 participants, 58 (4.4%) were excluded: 5 (0.4%) due to participants not being released from prison during the study period and 53 (4.0%) due to missing data on postcode of residence. The remaining 1267 (95.6%) participants were included in analyses. 

Descriptive statistics stratified by remoteness and area disadvantage are presented in Table 1. The mean age (±standard deviation) of the participants was 32.8 (±11.1) years. The majority of participants were male (*n* = 992; 78.3%) and 328 (25.9%) identified as Indigenous. Chi-squared tests indicated that participants who reported that they would be residing in remote locations after release from prison were disproportionately Indigenous men with a history of injecting drug use (IDU) and juvenile detention, and who had no stable employment and had a history of hospitalisation before incarceration. Participants who reported that they would reside in severely disadvantaged areas after release from prison were disproportionately Indigenous, had less than 10 years of schooling, had a history of IDU, and were nicotine dependent.

Crude incidence rates of hospitalisation in the cohort, stratified by remoteness and area disadvantage, are presented in Table 2. Eighty-two hospitalisations were excluded because they occurred during periods of reincarceration following the initial release from prison. Over a median (interquartile range) of 934 (718–1139) days of follow-up, 1199 hospitalisations occurred over a total of 3091 person-years of follow-up. The crude incidence rate (IR) of hospitalisation in the community was 388 (95%CI = 366–411) per 1000 person-years. We observed the highest crude incidence rates amongst people living in areas characterised by remoteness and area disadvantage (IR = 649; 95%CI: 526–791). The crude incidence rate was higher amongst individuals who experienced remoteness only (IR = 420; 95%CI: 349–501), than among those who experienced severe AD only (403, 95%CI = 351–461), and lowest among those who experienced neither remoteness nor AD (crude incidence rate 361, 95%CI: = 336–388) (Table 2).

Results from three modified Cox regression analyses are presented in Table 3. Unadjusted analyses indicated that remoteness (HR = 1.32, 95%CI = 1.04–1.69; *p* = 0.024) was associated with increased risk of hospitalisation after release from prison. A non-significant trend was observed for the association between area disadvantage (HR = 1.23, 95%CI = 0.98–1.54; *p* = 0.073) and increased risk of hospitalisation. After adjusting for covariate effects, the association between remoteness and hospitalisation after release from prison was attenuated to the null. Being Indigenous, being older, being female, having a high level of nicotine dependence, having been hospitalised prior to incarceration, and having poorer physical health were positively associated with hospitalisation after release from prison. A non-significant trend was observed for the association between injecting drug use and increased risk of hospitalisation (HR = 1.14, 95%CI = 0.91–1.43). Being employed prior to incarceration was a significant protective factor. A non-significant trend was observed for the association between years of schooling and decreased hospitalisation after release from prison (HR = 0.89, 95%CI = 0.70–1.14). Testing of interactions between Indigenous status, area disadvantage and remoteness indicated that Indigenous status did not significantly modify the effect of remoteness or area disadvantage on risk of hospitalisation. There was a non-significant trend of area disadvantage modifying the effect of remoteness on risk of hospitalisation (HR = 1.48, 95%CI = 0.90–2.45). 

Table 4 shows the five most common causes of hospitalisation in the cohort, and reports number of hospitalisations and total bed days. Injury, poisoning and certain other consequences of external causes were the most common reasons for hospitalisation (325 hospitalisations, 1003 bed days), followed by mental or behavioural disorders (217 hospitalisations, 1122 bed days).

## 4. Discussion

The aim of this study was to examine the relationship between social-environmental factors and hospitalisation in a cohort of adults released from prisons in Queensland, Australia. Findings supported the hypothesis that the incidence of hospitalisation would be higher in those returning to more remote and more socio-economically disadvantaged areas, and that remoteness was associated with increased risk of hospitalisation after release from prison. The effect on hospitalisation was compounded for individuals who experienced both remoteness and area disadvantage. 

We observed a significant difference in incidence rates of hospitalisation according to remoteness and area disadvantage, which is consistent with previous studies in the general population (for example, see [57,58]). Although social–environmental factors such as remoteness and area disadvantage are complex and may be closely linked with other social determinants of health, causal pathways are plausible. In addition, prior research has demonstrated that broader factors related to individual agency and the structural context in which people operate also contribute to poorer health outcomes in remote regions of Australia [59]. Individual needs are influenced by remoteness, and the extent to which these needs can be met is influenced by geographic location and broader social, economic and political structures.

In whole population studies, remoteness and area disadvantage have been associated with lower rates of primary care utilisation and higher rates of hospitalisation, which may in part be attributed to geographical barriers resulting in poorer access to services [60,61,62,63,64]. Recent reports in Australia have shown that remote areas are characterised by a lack of general practitioners [65], mental health services [34], and alcohol and other drug services [66]. Further, primary care utilisation is significantly lower amongst people who experience remoteness and severe area disadvantage compared to those who experience just one of these, indicating a compounding effect consistent with our findings [61,62,63,64,67,68,69,70]. Previous studies have demonstrated that contact with primary care services shortly after release from prison has the potential to increase primary care utilisation as individuals transition to the community [64,69]; however, this is also associated with increased risk of hospitalisation and it remains unknown if this was due to improved health management (i.e., people being hospitalised when needed) rather than an increase in avoidable hospitalisations [69]. Improving access to primary care in rural and remote areas may therefore be important for preventing avoidable hospitalisations, which may disproportionately affect disadvantaged populations such as ex-prisoners. There is a need for further research to disaggregate these findings by reason for hospitalisation (preventable vs. not) and by area disadvantage [64,69]. Such analysis, however, would require a much larger cohort, which may be difficult to achieve. 

Severely disadvantaged areas may offer poorer access of services that promote good health, due to an uneven distribution of health services, such as general practitioners and specialist services [71]. Additionally, severely disadvantaged areas may offer services that do not appropriately cater to the needs of people living in these areas. As a further potential causal pathway, stigma associated with disadvantage may have the effect of limiting opportunities for ex-prisoners in a specific area [21]. People may be reluctant to live in or do business in these areas, thus limiting opportunities for employment, which current findings suggest is protective against hospitalisation. Further, stigma associated with disadvantage has been found to affect a person’s sense of identity, achievement potential and self-worth [21]. This may lead to increased mental disorders and hence hospitalisations for mental and behavioural disorders, major causes of hospitalisation for the current study. As incarceration itself carries a stigma which may extend to communities characterised by high rates of incarceration, policy-makers may need to actively improve opportunities in disadvantaged areas in order to encourage successful transition from prison to the community.

A possible causal relationship between remoteness and hospitalisation after release from prison is that people in rural and remote areas may be disproportionately affected by social determinants of ill-health. Accordingly, we found that both lower levels of education and a lack of employment attenuated the association between remoteness and hospitalisation. Prior research has shown that lower levels of education and under-employment are more common in remote areas [72,73] and may play a role in shaping health outcomes [67,70]. Our findings indicate that under-employment was associated with risk of hospitalisation. Prior research in Australia and internationally has found under-employment to be associated with poor mental health outcomes [74]. Thus, increasing employment opportunities for people released from prison, particularly those with a lower level of education, may be a key intervention to prevent certain mental health hospitalisations. Additionally, rural and remote areas may be characterised by poorer access to services that improve health outcomes, including domestic violence response and emergency housing services [64,70]. The current findings suggest that injury and poisoning is the most common reason for hospitalisation amongst people recently released from prison, and hence improving access to services such as these may reduce the number of hospitalisations for preventable conditions or conditions for which primary care or community services could provide treatment.

Another possible explanation of the observed association between remoteness and hospitalisations in the present study relates to the Rural and Remote Medical Benefits Scheme (RRMBS), which allows people in Australia to receive bulk-billed primary care at specific hospitals in regional and remote Queensland. Doctors providing primary care in these hospitals may be more likely to admit patients as they are already at hospital. 

To the extent that the observed associations between remoteness and area disadvantage and hospitalisation are causal, our findings have important implications for the implementation of prisoner re-entry programs (particularly those focusing on housing assistance), which should consider the effects of both remoteness and area disadvantage as distinct yet potentially compounding environmental exposures that may increase the risk of hospitalisation. Previous research found that severe area disadvantage did not independently increase risk of violent recidivism [75], yet no previous research has looked at the link between area disadvantage and risk of hospitalisation despite the poor health outcomes of this population. 

In the unadjusted analyses, we found that remoteness (significant) and severe area disadvantage (non-significant) were associated with a 32% and 23% increase in risk of hospitalisation, respectively; however, these associations attenuated to the null after adjustment for covariates. This may indicate that the associations between social–environmental factors and hospitalisation were confounded by covariates, or, as mentioned above, that our covariates mediated these relationships. In adjusted analyses, we found that the risk of hospitalisation was greater for participants who were female, older and who had been hospitalised prior to incarceration. Being employed prior to incarceration was protective against hospitalisation. In contrast to previous research [3], neither stable accommodation nor education was protective against hospitalisation for people released from prison. No increased risk of hospitalisation was observed for Indigenous participants following adjustment, despite a previous finding that Indigenous people released from prison experience poorer health outcomes than non-Indigenous people released from prison [27,31]. This may be because of the strong association between Indigenous status and other measured covariates, such as remoteness. Our findings indicate that adults transitioning from prison to the community may benefit from re-entry programs that focus on securing employment and providing support for those with a history of hospitalisation, at least in respect to decreasing the risk of hospitalisation. Services that cater to the needs of female ex-prisoners may be particularly important, as current findings suggest risk of hospitalisation is significantly greater amongst females.

Our study is the first to examine the role of social–environmental factors in predicting hospitalisation among people recently released from prison. Important strengths of the study include the large cohort size, the representativeness of the study group (except for females being intentionally over-sampled), the prospective design, the combined use of rich baseline data and linked administrative data to determine hospitalisations, incarcerations and deaths and the use of ARIA [48] and SEIFA [49] datasets for information on remoteness and area disadvantage.

Our study has some limitations worth considering. We relied on self-reported postcode data at baseline to define geographic areas, thus we are assuming individuals moved to and remained in the postcodes they had identified prior to release. High rates of geographic mobility are common in disadvantaged groups, particularly where rates of homelessness are high [3]. However, in many instances, individuals move out of necessity, and are likely to move within area disadvantage categories rather than across them [3,76]. Furthermore, postcodes cover large and potentially diverse areas. In many cases, the area disadvantage score given to a postcode is an imperfect reflection of the level of socioeconomic disadvantage for all areas within that postcode [77]. Small, concentrated areas of disadvantage (‘disadvantage hotspots’) may not be accurately characterised as their level of disadvantage is below other areas within that same postcode. This inaccuracy has been observed in previous studies [76], and would tend to attenuate any association between area disadvantage and health outcomes. Future research may benefit from applying GIS mapping techniques to identify smaller geographical areas. Additionally, prior research has indicated that the intervention administered to one half of the cohort as part of an RCT increased self-reported access to primary care and ambulatory mental health services [4]. However, we found no association between intervention group and hospitalisation, suggesting that the case management intervention had no significant impact on patterns of hospitalisation in the cohort. Further research, evaluating the relationship between primary healthcare and hospitalisation amongst people released from prison, may be useful for informing service provision for those transitioning from prison to the community. Lastly, we did not measure the relationship between primary healthcare and hospitalisations in respect to social–environmental factors. Future studies aimed at examining the relationship between primary care and hospitalisations after release from prison, particularly hospitalisations for so-called ambulatory care sensitive conditions are warranted [12].

## 5. Conclusions

Among people released from prison, the rate of hospitalisation is higher in those who live in remote or socio-economically disadvantaged areas, although remoteness and area disadvantage were not found to be independently associated with risk of hospitalisation. An improved understanding of the relationship between social–environmental factors and health outcomes for people transitioning from prison to the community may inform improvements to re-entry programs for these vulnerable individuals.

## Figures and Tables

**Table 1:** Characteristics of the sample at baseline, according to remoteness and area disadvantage.

Characteristic		Total Observations (*n* = 1267)	Remoteness: Outer Regional, Remote and Very Remote (*n* = 185)	Remoteness: Inner Regional and Major Cities (*n* = 1087)	*p*-Value ^1^	AD ^2^: Severe (*n* = 296)	AD: Not Severe (*n* = 976)	*p*-Value ^1^
Socio-demographics	Female	21.7%	27.3%	20.7%	*p* = 0.045	23.9%	21.1%	*p* = 0.301
	Age (mean ± SD ^3^)	32.8 ± 11.1	31.7 ± 10.1	32.9 ± 11.3	*p* = 0.169 ^4^	32.0 ± 10.9	33.0 ± 11.2	*p* = 0.206 ^4^
	Indigenous	25.9%	59.5%	20.0%	*p* < 0.001	40.2%	21.5%	*p* < 0.001
	Employed prior to incarceration	50.8%	43.7%	52.0%	*p* = 0.035	47.0%	51.9%	*p* = 0.137
	Stable accommodation prior to incarceration	83.3%	86.6%	82.7%	*p* = 0.182	89.7%	81.3%	*p* = 0.001
	Completed at least 10 years schooling prior to incarceration	56.6%	52.6%	57.3%	*p* = 0.228	51.4%	58.2%	*p* = 0.037
Health-related characteristics	Has used injecting drugs	56.1%	36.7%	59.4%	*p* < 0.001	50.9%	57.6%	*p* = 0.039
	Hospitalised prior to being incarcerated	67.5%	76.3%	65.9%	*p* = 0.005	70.3%	66.6%	*P* = 0.238
	High smoking dependence	21.7%	18.4%	22.3%	*p* = 0.235	17.2%	23.1%	*p* = 0.033
	Scored above cohort median for PAM ^5^	50.2%	53.7%	49.6%	*p* = 0.303	47.0%	51.2%	*p* = 0.198
	Level of social support score (mean ± SD)	23.4 ± 6.6	22.7 ± 6.3	23.5 ± 6.6	*p* = 0.138 ^4^	24.0 ± 6.1	23.2 ± 6.7	*p* = 0.0823 ^4^
	Physical health score (mean ± SD)	54.2 ± 9.2	54.0 ± 8.7	54.2 ± 9.2	*p* = 0.750 ^4^	54.2 ± 9.4	54.15 ± 9.07	*p* = 0.889 ^4^
	Mental health score (mean ± SD)	44.1 ± 13.0	43.7 ± 12.8	44.2 ± 13.0	*p* = 0.623 ^4^	45.1 ± 12.6	43.8 ± 13.1	*p* = 0.137 ^4^
Justice-related characteristics	Has experienced juvenile detention	28.5%	37.4%	27.0%	*p* = 0.003	32.4%	27.4%	*p* = 0.090

^1^ Probability determined by interdependent samples *t*-test; ^2^ AD: Area disadvantage; ^3^ SD: Standard deviation; ^4^ Probability determined by Chi-square test for continuous variables; ^5^ PAM: Patient Activation Measure.

**Table 2:** Crude incidence rate (IR) of hospitalisation according to remoteness and area disadvantage.

		Remoteness IR ^1^ (95%CI)		Total
		Major Cities and Inner-Regional Areas	Outer Regional, Remote and Very Remote Areas	
Area disadvantage IR (95%CI)	Severe	403.4 (351.2–461.0)	648.7 (526.0–791.3)	457.1 (407.8–510.7)
	Not severe	361.1 (336.0–387.7)	419.8 (349.2–500.6)	368.3 (344.5–393.4)
	Total	369.6 (346.8–393.5)	496.7 (433.4–566.7)	387.9 (366.3–410.5)

^1^ Incidence rate.

**Table 3:** Risk of hospitalisations—univariate, multivariable and multivariable with interactions.

Variable	Model 1: Univariate Analysis		Model 2: Multivariable Analysis		Model 3: Multivariable Analysis with Interactions	
	Hazard Ratio	95%CI	Hazard Ratio	95%CI	Hazard Ratio	95%CI
Rural or remote	1.32	1.04–1.69	1.13	0.87–1.48	0.92	0.61–1.39
Severe area disadvantage	1.23	0.98–1.54	1.19	0.96–1.49	1.10	0.84–1.46
Female	1.83	1.51–2.23	1.55	1.26–1.91	1.54	1.25–1.90
Age	1.01	1.01–1.02	1.01	1.01–1.02	1.01	1.01–1.02
Indigenous	1.39	1.11–1.73	1.14	0.88–1.48	1.13	0.83–1.56
Employed prior to incarceration	0.63	0.52–0.76	0.80	0.65–0.98	0.79	0.65–0.97
Stable accommodation prior to incarceration	0.80	0.62–1.04	0.89	0.70–1.14	0.90	0.70–1.15
Completed at least 10 years of schooling at baseline	0.85	0.70–1.03	0.88	0.72–1.08	0.88	0.72–1.07
Has used injecting drugs	1.20	0.99–1.45	1.14	0.91–1.43	1.14	0.90–1.44
Hospitalised prior to being incarcerated	3.09	2.45–3.90	2.52	1.99–3.19	2.50	1.97–3.18
High smoking dependence	1.20	1.00–1.58	1.11	0.88–1.40	1.12	0.89–1.42
Scored above cohort median for PAM	1.07	0.88–1.30	1.14	0.93–1.38	1.13	0.93–1.37
Level of social support score	0.99	0.97–1.00	0.99	0.98–1.01	0.99	0.98–1.01
Physical health score	0.99	0.98–0.99	1.00	0.99–1.01	1.00	0.99–1.01
Mental health score	0.99	0.99–1.00	1.00	0.99–1.00	1.00	0.99–1.00
Has experienced juvenile detention	1.05	0.83–1.34	1.02	0.79–1.31	1.02	0.79–1.31
Indigenous status *^,1^ Remoteness	-	-	-	-	1.15	0.68–1.95
Indigenous status * Area disadvantage	-	-	-	-	1.07	0.67–1.72
Area disadvantage * Remoteness	-	-	-	-	1.48	0.90–2.45

^1^ *: Test for statistical interaction.

**Table 4:** Leading reasons for hospitalisation after release from prison, stratified by remoteness and area disadvantage.

Primary Diagnostic Category (ICD-10 Chapter)	Total		Rural or Remote, Severe Area Disadvantage (*n* = 64, 5.05%)		Not Rural or Remote, Severe Area Disadvantage (*n* = 229, 18.07%)		Rural or Remote, Not Severe Area Disadvantage (*n* = 123, 9.71%)		Not Rural or Remote, Not Severe Area Disadvantage (*n* = 851, 67.17%)	
	Hospitalisations (%)	Number of Bed Days (%)	Hospitalisations (%)	Number of Bed Days (%)	Hospitalisations (%)	Number of Bed Days (%)	Hospitalisations (%)	Number of Bed Days (%)	Hospitalisations (%)	Number of Bed Days (%)
Injury, poisoning and certain other consequences of external causes (XIX)	325 (27.1)	1003 (24.0 )	27 (27.8)	64 (19.3)	56 (26.4)	179 (23.7)	28 (22.4)	70 (19.5)	214 (28.1)	690 (25.3)
Mental and behavioural disorders (V)	217 (18.1)	1122 (26.8)	11 (11.3)	17 (5.1)	46 (21.4)	184 (24.3)	23 (18.4)	133 (37.0)	137 (18.0)	788 (28.8)
Diseases of the digestive system (XI)	86 (7.2)	197 (4.7)	9 (9.3)	36 (10.8)	11 (5.1)	23 (3.0)	16 (12.8)	27 (7.9)	50 (6.6)	110 (4.0)
Symptoms, signs and abnormal clinical and laboratory findings, not elsewhere classified (XVIII)	75 (6.3)	107 (2.6)	4 (4.1)	10 (3.0)	11(5.1)	13 (1.7)	13(10.4)	20 (5.6)	47 (6.2)	64 (2.3)
Diseases of the skin and subcutaneous tissue (XII)	62 (5.2)	189 (4.5)	5 (5.2)	18 (85.4)	11 (5.1)	30 (4.0)	4 (3.2)	10 (2.8)	42 (5.5)	131 (4.8)

## References

[1] Avery A., Kinner S.A. (2015). A robust estimate of the number and characteristics of persons released from prison in Australia. Aust. N. Z. J. Public Health.

[2] Walmsley R. (2015). World Prison Population List.

[3] Baldry E., McDonnell D., Maplestone P., Peeters M. (2006). Ex-Prisoners, homelessness and the state in Australia. Aust. N. Z. J. Criminol..

[4] Kinner S.A., Alati R., Longo M., Spittal M.J., Boyle F.M., Williams G.M., Lennox N.G. (2016). Low-intensity case management increases contact with primary care in recently released prisoners: A single-blinded, multisite, randomised controlled trial. J. Epidemiol. Community Health.

[5] Lovell D., Gagliardi G.J., Peterson P.D. (2002). Recidivism and use of services among persons with mental illness after release from prison. Psychiatr. Serv..

[6] Thomas E.G., Spittal M.J., Heffernan E.B., Taxman F.S., Alati R., Kinner S.A. (2016). Trajectories of psychological distress after prison release: Implications for mental health service need in ex-prisoners. Psychol. Med..

[7] Alan J., Burmas M., Preen D., Pfaff J. (2011). Inpatient hospital use in the first year after release from prison: A Western Australian population-based record linkage study. Aust. N. Z. J. Public Health.

[8] Fazel S., Baillargeon J. (2011). Review: The health of prisoners. Lancet.

[9] Snow K.J., Young J.T., Preen D.B., Lennox N.G., Kinner S.A. (2014). Incidence and correlates of hepatitis C virus infection in a large cohort of prisoners who have injected drugs. BMC Public Health.

[10] Van Dooren K., Richards A., Lennox N., Kinner S.A. (2013). Complex health-related needs among young, soon-to-be-released prisoners. Health Justice.

[11] Kinner S.A., Preen D.B., Kariminia A., Butler T., Andrews J.Y., Stoové M., Law M. (2011). Counting the cost: Estimating the number of deaths among recently released prisoners in Australia. Med. J. Aust..

[12] Wang E.A., Wang Y., Krumholz H.M. (2013). A high risk of hospitalization following release from correctional facilities in medicare beneficiaries: A retrospective matched cohort study, 2002 to 2010. JAMA Intern. Med..

[13] Cutcher Z., Degenhardt L., Alati R., Kinner S.A. (2014). Poor health and social outcomes for ex-prisoners with a history of mental disorder: A longitudinal study. Aust. N. Z. J. Public Health.

[14] Barnett A.G., Williams G.M., Schwartz J., Best T.L., Neller A.H., Petroeschevsky A.L., Simpson R.W. (2006). The Effects of Air Pollution on Hospitalizations for Cardiovascular Disease in Elderly People in Australian and New Zealand Cities. Environ. Health Perspect..

[15] Garden F.L., Jalaludin B.B. (2009). Impact of urban sprawl on overweight, obesity, and physical activity in Sydney, Australia. J. Urban Health.

[16] Sugiyama T., Leslie E., Giles-Corti B., Owen N. (2008). Associations of neighbourhood greenness with physical and mental health: Do walking, social coherence and local social interaction explain the relationships?. J. Epidemiol. Community Health.

[17] Mills V., Hooff M., Baur J., McFarlane A. (2012). Predictors of mental health service utilisation in a non-treatment seeking epidemiological sample of Australian adults. Community Ment. Health J..

[18] Australian Bureau of Statistics (2012). The Health and Welfare of Australia’s Aboriginal and Torres Strait Islander Peoples, Oct 2010.

[19] Pink B. (2011). Technical Paper: Socio-Economic Indexes for Areas (SEIFA).

[20] Krieger J., Higgins D.L. (2002). Housing and health: Time again for public health action. Am. J. Public Health.

[21] Clear T.R., Rose D.R., Ryder J.A. (2001). Incarceration and the community: The problem of removing and returning offenders. Crime Delinquency.

[22] Hayman N.E., White N.E., Spurling G.K. (2009). Improving Indigenous patients’ access to mainstream health services: The Inala experience. Med. J. Aust..

[23] Australian Bureau of Statistics (2014). Australian Aboriginal and Torres Strait Islander Health Survey: First Results, Australia, 2012–2013.

[24] Australian Institute of Health and Welfare (2014). Chronic Disease: Australia’s Biggest Health Challenge.

[25] Australian Bureau of Statistics (2013). Life Tables for Aboriginal and Torres Strait Islander Australians, 2010–2012.

[26] Australian Bureau of Statistics (2016). Prisoners in Australia, 2016.

[27] Lloyd J.E., Delaney-Thiele D., Abbott P., Baldry E., McEntyre E., Reath J., Indig D., Sherwood J., Harris M.F. (2015). The role of primary health care services to better meet the needs of Aboriginal Australians transitioning from prison to the community. BMC Fam. Pract..

[28] Australian Institute of Health and Welfare (2016). Australia’s Health 2016.

[29] Stewart L.M., Henderson C.J., Hobbs M.S.T., Ridout S.C., Knuiman M.W. (2004). Risk of death in prisoners after release from jail. Aust. N. Z. J. Public Health.

[30] Baldry E., Dowse L., Clarence M. (2012). People with Intellectual and Other Cognitive Disability in the Criminal Justice System.

[31] Lloyd J., Joshi C., Baldry E., McEntyre E., Indig D., Harris M., Thiele D., Mandjalan K.M., Abbott P., Reath J. (2013). The Effectiveness of Primary Health Care and Social Support Services in Meeting the Needs of Aboriginal People Released from the Criminal Justice System: A Systematic Literature Review from the SPRINT Project.

[32] Hunter E. (2007). Disadvantage and discontent: A review of issues relevant to the mental health of rural and remote Indigenous Australians. Aust. J. Rural Health.

[33] Australian Bureau of Statistics (2007). National Survey of Mental Health and Wellbeing: Summary of Results.

[34] National Rural Health Alliance Inc. (2017). Mental Health in Rural and Remote Australia.

[35] Griffiths K.M., Christensen H., Jorm A.F. (2009). Mental health literacy as a function of remoteness of residence: An Australian national study. BMC Public Health.

[36] Baldry E., McDonnell D., Maplestone P., Peeters M. (2003). Ex-Prisoners and Accommodation, What Bearing Do Different Forms of Housing Have on Social Reintegration?.

[37] Rachele J.N., Wood L., Nathan A., Giskes A., Turrell G. (2016). Neighbourhood disadvantage and smoking: Examining the role of neighbourhood-level psychosocial characteristics. Health Place.

[38] Sugiyama T., Villanueva K., Knuiman M., Francis J., Foster S., Wood L., Giles-Cortii B. (2016). Can neighborhood green space mitigate health inequalities? A study of socio-economic status and mental health. Health Place.

[39] Banham D., Chen T., Karnon J., Brown A., Lynch J. (2017). Sociodemographic variations in the amount, duration and cost of potentially preventable hospitalisation for chronic conditions among Aboriginal and non-Aboriginal Australians: A period prevalence study of linked public hospital data. Health Serv. Res..

[40] Kinner S.A., Lennox N., Williams G.M., Carroll M., Quinn B., Boyle F.M., Alati R. (2013). Randomised controlled trial of a service brokerage intervention for ex-prisoners in Australia. Contemp. Clin. Trials.

[41] Kinner S.A., van Dooren K., Boyle F.M., Longo M., Lennox N. (2014). Development of an intervention to increase health service utilisation in ex-prisoners. Health Justice.

[42] Optum SF-36v2 Health Survey. http://campaign.optum.com/content/optum/en/optum-outcomes/what-we-do/health-surveys/sf-36v2-health-survey.html.

[43] National Institute on Drug Abuse Instrument: Fagerstrom Test for Cicotine Dependence. https://datashare.nida.nih.gov/instrument/fagerstrom-test-for-nicotine-dependence.

[44] Hibbard H.H., Stockard J., Mahoney E.R., Tusler M. (2004). Development of the Patient Activation Measure (PAM): Conceptualizing and measuring activation in patients and consumers. Health Serv. Res..

[45] Collaborative Studies Coordinating Center (2003). ENRICHD Social Support Instrument (ESSI).

[46] National Centre for Special Aplications of GIS ARIA+ 2011. https://www.adelaide.edu.au/hugo-centre/spatial_data/.

[47] Clark R.A., Coffee N., Turner D., Eckert K.A., van Gaans D., Wilkinson D., Stewart S., Tonkin A.M. (2012). Application of geographic modeling techniques to quantify spatial access to health services before and after an acute cardiac event: The Cardiac Accessibility and Remoteness Index for Australia (ARIA) project. Circulation.

[48] Australian Bureau of Statistics (2013). Australian Statistical Geography Standard (ASGS): Volume 5—Remoteness Structure, July 2011.

[49] Australian Bureau of Statistics (2013). Census of Population and Housing: Socio-Economic Indexes for Areas (SEIFA), Australia, 2011.

[50] Kavanagh A.M., Goller J.L., King T., Jolley D., Crawford D., Turrell G. (2005). Urban area disadvantage and physical activity: A multilevel study in Melbourne, Australia. J. Epidemiol. Community Health.

[51] Queensland Health Queensland Hospital Admitted Patient Data Collection (QHAPDC). https://www.health.qld.gov.au/hsu/collections/qhapdc.

[52] Australian Institute of Health and Welfare (AIHW) National Death Index (NDI). https://www.aihw.gov.au/about-our-data/our-data-collections/national-death-index/about-national-death-index.

[53] Larney S., Burns L. (2011). Evaluating health outcomes of criminal justice populations using record linkage: The importance of aliases. Eval. Rev..

[54] Queensland Corrective Services Department of Justice and Attorney-General. https://www.qld.gov.au/law/sentencing-prisons-and-probation/data-and-research.

[55] Andersen P.K., Gill R.D. (1982). Cox’s regression model for counting processes: A large sample study. Ann. Stat..

[56] National Centre for Classification in Health (2013). The International Statistical Classification of Diseases and Related Health Problems: 10th Revision: Australian Modification (ICD-10-AM).

[57] Banham D., Woollacott T., Gray J., Humphrys B., Mihnev A., McDermott R. (2010). Recognising potential for preventing hospitalisation. Aust. Health Rev..

[58] Glover J. (2007). Avoidable hospitalisations—A national map of who is affected. Health Voices.

[59] Bourke L., Humphreys J.S., Wakerman J., Taylor J. (2012). Understanding rural and remote health: A framework for analysis in Australia. Health Place.

[60] Eckert K.A., Taylor A.W., Wilkinson D. (2004). Does health service utilisation vary by remoteness? South Australian population data and the Accessibility and Remoteness Index of Australia. Aust. N. Z. J. Public Health.

[61] Einarsdóttir K., Preen D.B., Emery J.D., Kelman C., Holman C.D.A.J. (2010). Regular primary care lowers hospitalisation risk and mortality in seniors with chronic respiratory diseases. J. Gen. Intern. Med..

[62] Turrell G., Oldenburg B.F., Harris E., Jolley D. (2004). Utilisation of general practitioner services by socio-economic disadvantage and geographic remoteness. Aust. N. Z. J. Public Health.

[63] Ansari Z., Haider S.I., Ansari H., de Gooyer T., Sindall C. (2012). Patient characteristics associated with hospitalisations for ambulatory care sensitive conditions in Victoria, Australia. BMC Health Serv. Res..

[64] Wang E.A., Hong C.S., Shavit S., Sanders R., Kessell E., Kushel M.B. (2012). Engaging individuals recently released from prison into primary care: A randomized trial. Am. J. Public Health.

[65] Duckett S., Breadon P. (2013). Access All Areas: New Solutions for GP Shortages in Rural Australia.

[66] National Rural Health Alliance Inc. (2014). Alcohol Use in Rural Australia.

[67] Wainer J., Chesters J. (2000). Rural mental health: Neither romanticism nor despair. Aust. J. Rural Health.

[68] Page A., Ambrose S., Glover J., Hetzel D. (2007). Atlas of Avoidable Hospitalisations in Australia: Ambulatory Care-Sensitive Conditions.

[69] Young J.T., Arnold-Reed D., Preen D., Bulsara M., Lennox N., Kinner S.A. (2015). Early primary care physician contact and health service utilisation in a large sample of recently released ex-prisoners in Australia: Prospective cohort study. BMJ Open.

[70] National Rural Health Alliance Inc. (2014). Income Inequality Experienced by the People of Rural and Remote Australia.

[71] Australian Bureau of Statistics (2010). Australian Social Trends, Mar 2010.

[72] Australian Bureau of Statistics (2008). Australian Social Trends, 2008.

[73] Australian Bureau of Statistics (2016). National Aboriginal and Torres Strait Islander Social Survey, 2014–15.

[74] Hergenrather K.C., Zeglin R.J., McGuire-Kuletz M., Rhodes S.D. (2015). Employment as a social determinant of health: A review of longitudinal studies exploring the relationship between employment status and mental health. Rehabil. Res. Policy Educ..

[75] Sariaslan A., Larsson H., Lichtenstein P., Fazel S. (2017). Neighborhood Influences on Violent Reoffending Risk in Released Prisoners Diagnosed with Psychotic Disorders. Schizophr. Bull..

[76] Hyndman J.C., Holman C.D., Hockey R.L., Donovan R.J., Corti B., Rivera J. (1995). Misclassification of social disadvantage based on geographical areas: Comparison of postcode and collector’s district analyses. Int. J. Epidemiol..

[77] Kwan M. (2012). The uncertain geographic context problem. Ann. Assoc. Am. Geogr..

